# Frontal sinus approach in complicated chronic rhinosinusitis with nasal polyps: Our experience

**DOI:** 10.22088/cjim.14.4.746

**Published:** 2023

**Authors:** Maxim Aflitonov, Aleksei Voronov, Vladimir Dvoryanchikov, Sergei Artyushkin, Evgeniyav Bezrukova

**Affiliations:** 1 Immanuel Kant Baltic Federal University, Kaliningrad, Russia; 2 Saint-Petersburg Research Institute of Ear, Throat, Nose and Speech, Saint-Petersburg, Russia; 3 North-Western State Medical University named after I.I. Mechnikov, Saint-Petersberg, Russia

**Keywords:** Draf3, Modified lothrop procedure, Chronic rhinosinusitis, Frontoethmoidal area hyperostosis, Hyperostosis staging

## Abstract

**Background:**

Hyperostosis is a common consequence of chronic rhinosinusitis with nasal polyps (CRSwNP) and other chronic rhinosinusitis, affecting mainly structures of the ethmoid labyrinth, frontal and maxillary sinuses. These neo-bones are found in advanced stages of rhinosinusitis causes exacerbation because of ostia outflow blockage. Frequent relapses of the disease due to hyperostosis restenosis, represent a problem in the treatment of chronic frontal sinusitis. We report our cases experience about the treatment of frontal chronic sinusitis because of frontoethmoidal area hyperostosis, treated by modified lothrop procedure, achieving sufficient clinical effect.

**Case Presentation:**

A 52-year-old female (CRSwNP) with right side frontoethmoidal area totally obstructed by 25 ×15 ×10 mm (height-length-width) bony mass, with total opacification of the right frontal and left maxillary sinuses, right frontal sinus anterior wall defect. A 63-year-old male (CRSwNP) with a 23 ×10 ×10 mm (height-length-width) bony mass arising from the right frontoethmoidal area which causes total opacification of the right frontal sinus, inferior and posterior sinus wall defect.

**Conclusion:**

Modified lothrop procedure is a method of choice for frontoethmoidal bone hyperostosis surgery to be performed endoscopically. Endoscopy provides excellent visualization of anatomy and a better approach to bone hyperostosis zone.

Frequent relapses of the disease after surgical treatment, represent a problem in the treatment of chronic frontal sinusitis. Causes of chronic frontal sinusitis recurrence are well known. This is due to narrowing, scarring, mucosae overgrowth inside the frontal sinuses neo-ostium. Formation of the frontal sinuses neo-ostium is the most difficult stage of surgery on the frontal sinus (1). 

Functional endoscopic sinus surgery gives good long-term results, if the patient has a Khun 1-4 type cells. In chronic rhinosinusitis with or without nasal polyps, the frontoethmoidal area (Khun1- 4 cells and other) can be obstructed by progressive bone hyperostosis (inflammatory neo-bone growth). The new hyperostosis can completely obstruct the frontoethmoidal area for various lengths (2).

Frontoethmoidal region obstruction by bone tissue overgrowth is insufficiently described in the literature, there is no anatomical classification of bone overgrowth of the frontoethmoidal area, and how they affect the technique and long-term results of functional endoscopic surgery. We report our experience about the treatment of frontal mucoceles/ chronic sinusitis because of frontoethmoidal area hyperostosis, treated by modified lothrop procedure, achieving total removal of the neo- bone structures with sufficient clinical effect (3, 4).

Our staging intends to classify the severity and extent of a patient’s disease based on the measurable amount of frontoethmoidal area bone obstruction as a result of chronic sinusitis and to assess the specific factors that may contribute to the complexity of long-term case management (4).

## Case Presentation

This study was carried out in the school of surgery of Immanuel Kant Baltic Federal University, Russia, Kaliningrad. This study was approved by the Ethics Committee of State Budgetary Health Institution "Regional Clinical Hospital of the Kaliningrad Region" (ethics committee co: AB2345.2020).

**Figure 1:**
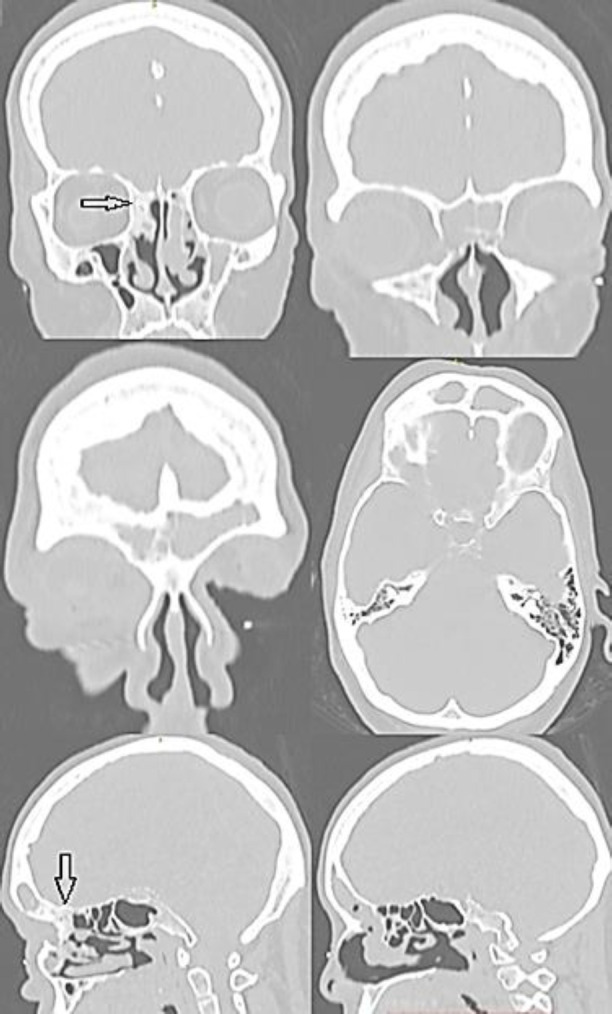
Paranasal sinuses CT scan showing a 25×15×10 mm (height-length-width) bony mass (black arrow) arising from the right frontoethmoidal area which causes total opacification of the right frontal sinus and right frontal sinus anterior wall defect with orbital cellulitis. Left maxillary sinus total opacification

Case 1: A 52-year-old female complained of swelling of the eye, headache, purulent nasal discharge, inability to open right eye for the past 5 days. She associated it with chronic rhinosinusitis with nasal polyps, but there was no history of sinus operation. The patient, already poorly treated by antibiotic therapy, after developing a cheek and eye swelling, came to our otorhinolaryngology department in January 2020. On examination: purulent discharge on both sides from the nose, cheek and eyes swelling on the right, swelling mucosa and polyps in the middle nasal meatus on both sides. Computed tomography (CT) scan of the paranasal sinuses showed a right side frontoethmoidal area totally obstructed by 25 × 15 × 10 mm bony mass, totally opacification of the right frontal and left maxillary sinuses, right frontal sinus anterior wall defect with orbital cellulitis (figure1).

**Figure 2:**
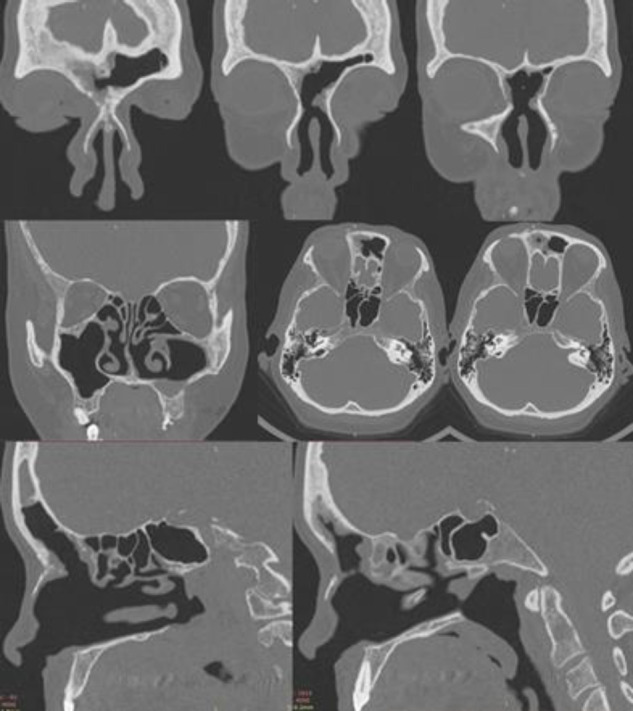
Paranasal sinuses two-month CT scan after operation (Modified lothrop procedure condition): free frontoethmoidal area with complete bilateral drainage, free inferior maxillary antrostomy with complete inferior meatus drainage

The surgery was performed with the patient under general anesthesia with the use of 0 °, and 70 ° telescopes via endonasal endoscopic approach with navigator support. The bony mass, frontal beak, partial ethmoidal cells adherent to the anterior cranial wall, superior part of the nasal septum were removed by drills 0 °, and 70 (Modified lothrop procedure), allowing the frontal sinuses to drain into the nose. Left maxillary sinus antrostomy with polyps and purulent discharge removal were performed. Given the cellulitis dimensions, an incision of the right orbital was performed to drain purulent discharge out. Right endonasal canthotomy was performed to drain orbital purulent discharge inside the nose. The mucosal and bony specimens from sinuses were submitted for histological evaluation, and diagnosed as bone/ mucosae. Weekly endoscopic control and physical examination were performed after surgery, with good patient clinical improvement, and strict compliance to irrigation and mometasone intranasal use for six months. Complete relief of her symptoms with no complications and/or surgical sequelae was in 14 days. Outpatient clinic provided endoscopic crusts removal from both nasal sides for 8 weeks. After surgery in two months, the patient provided a CT scan that showed complete removal of cellulitis and sinusitis. There was no total stenosis in frontoethmoidal area, or recurrence of sinusitis (figure 2) (5). 

**Figure 3:**
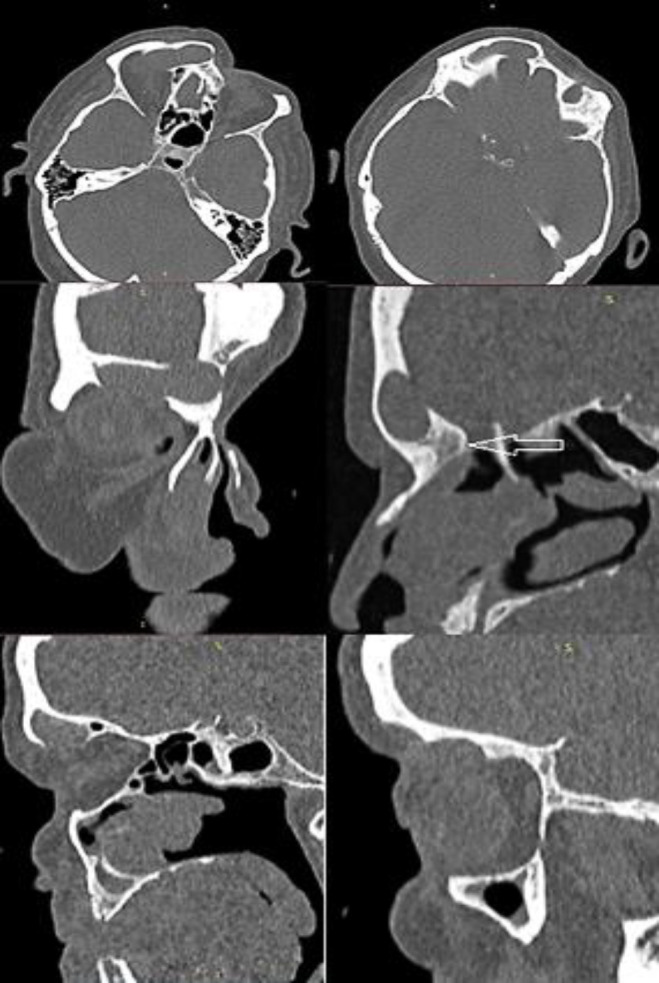
Paranasal sinus CT scan showing a 23×10×10 (height-length-width) mm bony mass (white arrow) arising from the right frontoethmoidal area which causes total opacification of the right frontal sinus, inferior and posterior sinus wall defect

Case 2: A 63-year-old male complained of intermittent localized pain over the right supraorbital area for the past nine months, not associated with fever or nasal discharge (fig. 1). There was no history of trauma. The patient already underwent a left Lynch-Howarth frontethmoidectomy in 1988 and bilateral maxillary sinus antrostomy, bilateral polypectomy because of chronic rhinosinusitis with nasal polyps/ left frontal sinus mucoceles. Outpatient clinic provided CT scan with detection of right frontal sinus mucoceles with destruction of anterior and posterior frontal sinus wall (figure 3). He came to our department in May 2020. 

**Figure4:**
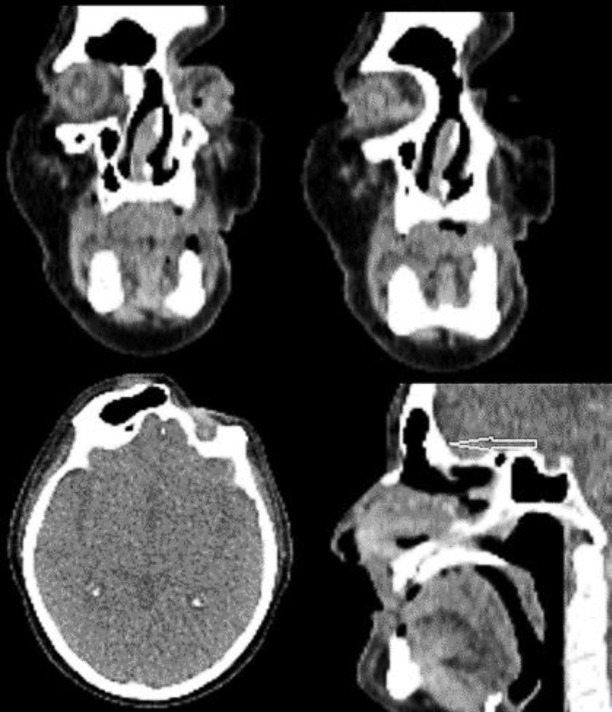
Paranasal sinus two-month CT scan after operation (Modified lothrop procedure condition): free frontoethmoidal area with complete right frontal drainage. Remnants of frontoethmoidal bone hyperostosis (white arrow) on anterior cranial fossa wall

Under general anesthesia with the use of 0 °, and 70 ° telescopes with navigator support was provided endonasal endoscopic approach. The bone neoformation was removed by drill. To drain frontal sinus into the nose Modified hemi- lothrop procedure was performed and histological evaluation was diagnosed as bone/ mucosae. Ethmoidal cells and the superior part of the nasal septum were also removed. Endoscopic nasal toilets in outpatient clinics were provided after surgery. Two months after surgery, the patient provided a CT scan that showed complete removal of sinus opacification. There was no total stenosis in the frontoethmoidal area (figure. 4) (6). 

## Discussion

Bone frontoethmoidal area hyperostosis in chronic sinus inflammation has been studied little in world literature. We have successfully operated dozens of cases over the past 10 years. We have shown clinical examples on the feasibility and successful result of the intranasal approach. The success of frontal sinus drainage directly depends on the anatomical structure of the frontoethmoidal region and the type of pathological process occurring in the nasal cavity and paranasal sinuses as a whole (7). The following landmarks are anatomically important: distance (width) from the beginning of the frontal beak to the beginning of the skull base of the anterior cranial fossa, the length (height) on which the frontal beak is located. The width and height of the frontal beak (assuming it is healthy) is irrelevant, as it can easily be removed to the outer skin. Obliteration degree of the specified width and height by the inflammatory hyperostosis neo-bone is of prime importance. In the drilling area of this bone, a new increased hyperostosis and subsequent stenosis occurs. In сhronic rhinosinusitis with nasal polyps, there is a progressive growth of neo-bony due to progressive inflammation and osteitis with new increased hyperostosis (8). In CRSwNP and isolated form of frontal sinusitis, the hyperostosis is much harder and more difficult amenable to postoperative hormone (mometasone) therapy (9). The last value is the angle of the frontal beak. We have collected all these values in the frontoethmoidal region bone hyperostosis staging (table 1). Modified lothrop procedure is a method of choice for frontoethmoidal bone hyperostosis surgery to be performed transnasally. Endoscopy provides excellent visualization of anatomy and a better approach to bone hyperostosis zone. 

**Table 1:** Frontoethmoidal region bone hyperostosis staging, surgeries results

	**Stage I**	**Stage II**	**Stage III**
**Patient amount**	16	17	23
**Extent and distribution**	width ≤15 mm height≤10 mm	width ≤10 mm height ≥10 mm	width ≤5 mm height ≥15 mm
**Fronto-nasal angle**	≤30° ≥30°	≤60° ≥60°	≤90° ≥90°
**Modified Lothrop procedure (endonasal)**	0-S; 9-F	1-S; 9-F	3-S; 11-F
**Externs l front ethmoidectomy**	4-S; 3-F	4-S; 3-F	8-S; 1-F

S: Stenosis; F: Free
